# Supplementary material for: Patient-Selection of a Clinical Trial Primary Outcome: The ENHANCE-AF Outcomes Survey
Source: PLoS One. 2025 Mar 7;20(3):e0318858. doi: 10.1371/journal.pone.0318858 (PMC11888126; doi:10.1371/journal.pone.0318858)
Supplement: S1 File — (PDF) [file pone.0318858.s001.pdf]

Screening

Record ID	______________________
Date consent obtained	______________________ (No study data should be collected prior to consent)
What language was the consent administered in?	◯ English<br>◯ Spanish
Name of study team member who administered consent	______________________ (Team member must be trained in administering consent.)
Participant First Name	______________________
Participant Last Name	______________________
Participant Study ID	______________________ (#-### (Site code - participant number starting at 001 and increasing by 1))
Participant's date of birth	______________________
Participant's gender	◯ Male<br>◯ Female<br>◯ Transgender Male<br>◯ Transgender Female<br>◯ Other<br>◯ Decline to State
What is the highest grade level of education achieved?	◯ Less than 6th grade<br>◯ 6th to 10th grade<br>◯ 11th to 12th grade<br>◯ Some college<br>◯ 2 - year degree<br>◯ 4 - year degree<br>◯ Post graduate<br>◯ Doctorate<br>◯ Decline to State
Does the participant currently take an anticoagulant?	◯ Yes ◯ No ◯ Unknown
If not currently taking, have you previously taken an anticoagulant?	◯ Yes ◯ No ◯ Unknown
Is the participant currently an active participant in any other research studies?	◯ Yes ◯ No<br>(If yes, confirm eligibility with study PIs)

If yes, does the participant have approval from other study team(s) to participate in this study?	◯ Yes ◯ No

Inclusion Criteria

Is the participant 18 years or older?	◯ Yes ◯ No
Is the participant diagnosed with Non-Valvular Atrial Fibrillation?	◯ Yes ◯ No
CHA2DS2-VASc Calculator (inclusion criteria is 3 for Female, 2 for Male)	
Does the participant have Congestive Heart Failure (CHF)?	◯ Yes ◯ No
Does the participant have High Blood Pressure (Hypertension)?	◯ Yes ◯ No
Is the participant 75 years or older?	◯ Yes ◯ No
Does the participant have Diabetes Mellitus (Diabetes)?	◯ Yes ◯ No
Does the participant have a history of stroke or TIA or thromboembolism?	◯ Yes ◯ No
Does the participant have Vascular Disease (previous MI, Coronary artery disease (CAD), Peripheral arterial disease (PAD), Aortic atherosclerosis, and Carotid artery disease)	◯ Yes ◯ No
Is the participant age 65 to 74 years old?	◯ Yes ◯ No
Does the participant identify as female?	◯ Yes ◯ No
CHA2DS2-VASc score (calculated)	________________________
CHA2DS2-VASc score eligibility (1 = eligible, 0 = not eligible)	________________________

Exclusion Criteria

Does the participant have a history of hemodynamically significant mitral stenosis or mechanical valve replacement?	◯ Yes ◯ No<br>(if 'yes' participant is NOT eligible to participate in ranking survey)
Does the participant have any absolute contraindication to anticoagulation?	◯ Yes ◯ No<br>(if 'yes' participant is NOT eligible to participate in ranking survey)
Has the participant declined to continue with the study?	◯ Yes ◯ No<br>(if 'yes' participant is NOT eligible to participate in ranking survey)

Eligibility for Survey

Is this person eligible to participate in the ranking survey?	◯ Yes ◯ No<br>(Please check all inclusion and exclusion criteria for accuracy)

Ranking

Record ID	______________________
What language was the survey presented in?	○ English ○ Spanish
What is the group identifier associated with this entry?	☐ M ☐ O

Please enter the appropriate rank for each list of questions.

	5-question survey	16-question survey
Rank 1 (Top Choice)	○	○
Rank 2	○	○

Naming

Record ID ______________________

We will be conducting a study to determine whether our new approach to educating patients about stroke prevention improves outcomes. We are asking patients to help us select a name for this study. Please check your favorite option from the 3 choices below.

◯ ENHANCE -AF: Engaging Patients to Help Achieve Increased Patient Choice and Engagement for AFib Stroke Prevention
◯ SAFE: Strategies for AFib Engagement in Decision Making for Anticoagulation
◯ ENGAGE: Enhancing Good Practices for Anticoagulation Guideline Based Treatment and Empowerment of Patients
