# Supplementary material for: Patient-Selection of a Clinical Trial Primary Outcome: The ENHANCE-AF Outcomes Survey
Source: PLoS One. 2025 Mar 7;20(3):e0318858. doi: 10.1371/journal.pone.0318858 (PMC11888126; doi:10.1371/journal.pone.0318858)
Supplement: Attachment — Submitted filename: PLOSONE Response to Reviewers_Nov26ER.docx [file pone.0318858.s002.docx]

November 26th, 2024

PONE-D-24-40722

**Patient-Selection of a Clinical Trial Primary Outcome: The ENHANCE-AF Outcomes Survey**

Dear Daniel Antwi-Amoabeng, MD, MSc,

Thank you very much for the supportive review of our manuscript. We appreciate the additional helpful suggestions from the editor and reviewers and have made the modifications listed below to improve the presentation of our analysis. We believe the manuscript is greatly improved as a result of these changes.

Again, we appreciate PLOS ONE’s support in the investigation of the importance of patient selection of a clinical trial primary outcome and believe this work will be of great interest to your readership.

All reviewer comments are listed in **bold**. Please find our responses below the reviewer’s comment.

Sincerely,

Randall S. Stafford, MD, PhD,

Professor of Medicine

Stanford University School of Medicine, Stanford Prevention Research Center

**REVIEWER COMMENTS AND MANUSCRIPT MODIFICATIONS**

**Reviewer #1: I applaud the authors for putting together an effective manuscript detailing the pretrial selection of an appropriate clinical tool to assess patient outcomes and in assessing the patient clinical comorbidity burden (CHADSVASC score) in relation to the preference of the particular questionnaire ( DCS vs DRS). I have some suggestions and recommendations as detailed below.**

1. **The authors describe a 15- minute long, seven sections and 34 questions long questionnaire: please include that as a supplement or a table to allow the readers an insight into assessment of patient preference.**

We appreciate the reviewer’s recommendation and have added the questionnaire in the Supplemental Material.

2. **Please describe/enlist components of the multivariate regression model utilized.**

We agree with the reviewer’s comment that it is important to describe regression model in greater detail and have done so within the manuscript’s statistical analysis section.

3. **Please restructure the conclusion and discussion section: the last 6-7 lines of conclusion can be elaborated on in the discussion for the ease of the readers.**

We appreciate the opportunity to provide greater depth to the manuscript and have revised the conclusion section to emphasize the importance of using patient collaboration in clinical trial design.

**Reviewer #2: In the current paper, the authors aimed to find the most appropriate, patient-selected primary outcome of the ENHANCE-AF clinical trial and to examine whether outcome selection differed by demographical and clinical characteristics. Patients with AF selected the DCS (77%) over the DRS (23%) to measure their decision-making experience with OAC for stroke prevention. The authors decided on the DCS as the primary endpoint for the ENHANCE-AF clinical trial. Patient knowledge and compliance are essential for the treatment and follow-up of the patients (patient-centeredness).**

1. **What about the impact of education on digital SDM tools to guide the use of OACs in stroke prevention for patients with AF?**

We evaluated the potential impact of educational level on the selection of DCS vs. DRS. We found no significant difference in the likelihood of favoring DCS between patients with a 2-year degree or less (79%) and those with a 4-year degree or more (79%), OR = 1.05, 95% CI: 0.35, 3.12, p = 1.00. This has been added into the text. Additionally, when included in the regression model, educational level did not emerge as a significant independent predictor of DCS preference.

2. **Furthermore, how will the authors solve the problem of patients with previous strokes who have difficulty understanding the SDM tool? Patients with a history of stroke should be evaluated separately if they have had difficulty with such tools.**

We appreciate the reviewer’s insightful comment here. We agree that future trials should evaluate patients with a history of stroke separately. However, the current informed consent process required a moderate level of cognitive ability for inclusion in the study. Thus, we did not include individuals impaired at the level implied by the reviewer.

3. **Furthermore, you can revise your analysis using the CHADS-VA score rather than the CHADS-VASc score, consistent with the recent AF guidelines**

While we are interested in the implication of dropping gender from CHADVasc2 to create better calibration of men and women’s scores (CHADS-VA), we have chosen to continue to employ CHADSVasc scores as a potentially moderating variable. While 2024 European Society of Cardiology AFib guidelines suggest switching to the gender-neutral CHADS-VA, the 2023 AHA/ACC guidelines continue to promote CHADSVasc2 (with gender as a scoring variable) as the recommended risk stratification tool.
